# Deep Learning With EEG Spectrograms in Rapid Eye Movement Behavior Disorder

**DOI:** 10.3389/fneur.2019.00806

**Published:** 2019-07-30

**Authors:** Giulio Ruffini, David Ibañez, Marta Castellano, Laura Dubreuil-Vall, Aureli Soria-Frisch, Ron Postuma, Jean-François Gagnon, Jacques Montplaisir

**Affiliations:** ^1^Neuroelectrics Corporation, Cambridge, MA, United States; ^2^Applied Neuroscience, Starlab Barcelona, Barcelona, Spain; ^3^Department of Neurology, Montreal General Hospital, Montreal, QC, Canada; ^4^Centre for Advanced Research in Sleep Medicine, Hôpital du Sacré-Cœur de Montréal, Montreal, QC, Canada; ^5^Department of Psychiatry, Université de Montréal, Montreal, QC, Canada

**Keywords:** PD, RBD, Deep learning, EEG, time-frequency analysis

## Abstract

REM Behavior Disorder (RBD) is now recognized as the prodromal stage of α-synucleinopathies such as Parkinson's disease (PD). In this paper, we describe deep learning models for diagnosis/prognosis derived from a few minutes of eyes-closed resting electroencephalography data (EEG) collected at baseline from idiopathic RBD patients (*n* = 121) and healthy controls (HC, *n* = 91). A few years after the EEG acquisition (4±2 years), a subset of the RBD patients were eventually diagnosed with either PD (*n* = 14) or Dementia with Lewy bodies (DLB, *n* = 13), while the rest remained idiopathic RBD. We describe first a simple deep convolutional neural network (DCNN) with a five-layer architecture combining filtering and pooling, which we train using stacked multi-channel EEG spectrograms from idiopathic patients and healthy controls. We treat the data as in audio or image classification problems where deep networks have proven successful by exploiting invariances and compositional features in the data. For comparison, we study a simple deep recurrent neural network (RNN) model using three stacked Long Short Term Memory network (LSTM) cells or gated-recurrent unit (GRU) cells—with very similar results. The performance of these networks typically reaches 80% (±1%) classification accuracy in the balanced HC vs. PD-conversion classification problem. In particular, using data from the best single EEG channel, we obtain an area under the curve (AUC) of 87% (±1%)—while avoiding spectral feature selection. The trained classifier can also be used to generate synthetic spectrograms using the *DeepDream* algorithm to study what time-frequency features are relevant for classification. We find these to be bursts in the theta band together with a decrease of bursting in the alpha band in future RBD converters (i.e., converting to PD or DLB in the follow up) relative to HCs. From this first study, we conclude that deep networks may provide a useful tool for the analysis of EEG dynamics even from relatively small datasets, offering physiological insights and enabling the identification of clinically relevant biomarkers.

## 1. Introduction

RBD is a parasomnia characterized by intense dreams with during REM sleep without muscle atonia (1), i.e., with vocalizations and body movements. Idiopathic RBD occurs in the absence of any neurological disease or other identified cause, is male-predominant and its clinical course is generally chronic progressive (2). Several longitudinal studies conducted in sleep centers have shown that most patients diagnosed with the idiopathic form of RBD will eventually be diagnosed with a neurological disorder such as Parkinson disease (PD) or dementia with Lewy bodies (DLB) (1–4). In essence, idiopathic RBD has been suggested as a prodromal stage of α-synucleinopathies [PD, DLB, and less frequently multiple system atrophy (MSA) (1, 4)].

RBD has an estimated prevalence of 15–60% in PD and has been proposed to define a subtype of PD with relatively poor prognosis, reflecting a brainstem-dominant route of pathology progression (see (5) and references therein) with a higher risk for dementia or hallucinations. PD with RBD is characterized by more profound and extensive pathology—not limited to the brainstem—, with higher synuclein deposition in both cortical and sub-cortical regions.

Electroencephalographic (EEG) and magnetoencephalographic (MEG) signals contain rich information associated with functional processes in the brain. To a large extent, progress in their analysis has been driven by the study of spectral features in electrode space, which has indeed proven useful to study the human brain in both health and disease. For example, the “slowing down” of EEG is known to characterize neurodegenerative diseases (6–8). It is worth mentioning that the selection of disease characterizing features from spectral analysis is mostly done after an extensive search in the frequency-channel domain.

However, neuronal activity exhibits non-linear dynamics and non-stationarity across temporal scales that cannot be studied properly using classical approaches. Tools capable of capturing the rich spatiotemporal hierarchical structures hidden in these signals are needed. In Ruffini et al. (8), for example, algorithmic complexity metrics of EEG spectrograms were used to derive information from the dynamics of EEG signals in RBD patients, with good results, indicating that such metrics may be useful *per se* for classification or scoring. However, ideally we would like to use methods where the relevant features are found directly by the algorithms.

Deep learning algorithms are designed for the task of exploiting compositional structure in data (9). In past work, for example, deep feed-forward autoencoders have been used for the analysis of EEG data to address the issue of feature selection, with promising results (10). Interestingly, deep learning techniques, in particular, and artificial neural networks in general are themselves bio-inspired by the brain—the same biological system generating the electric signals we aim to decode. This suggests they may be well suited for the task.

Deep recurrent neural networks (RNNs), are known to be potentially Turing complete [see, e.g., (11) for a review], but general RNN architectures are notoriously difficult to train (12). In this regard, it is worth mentioning that “reservoir” based RNN training approaches are evolving (13). In earlier work, a particular class of RNNs called Echo State Networks (ESNs) that combine the power of RNNs for classification of temporal patterns and ease of training (14) was used with good results with the problem at hand. The main idea behind ESNs and other “reservoir computation” approaches is to use semi-randomly connected, large, fixed recurrent neural networks where each node/neuron in the reservoir is activated in a non-linear fashion. The interior nodes with random weights constitute what is called the “dynamic reservoir” of the network. The dynamics of the reservoir provides a feature representation map of the input signals into a much larger dimensional space (in a sense much like a kernel method). Using such an ESN, an accuracy of 85% in a binary, class-balanced classification problem (healthy controls vs. PD patients) was obtained using a relatively small dataset in Ruffini et al. (14). The main limitations of this approach, in our view, are the computational cost of developing the reservoir dynamics of large random networks and the associated need for feature selection (e.g., which subset of frequency bands and channels to use as inputs to simplify the computational burden).

In this paper we use a similar but simpler strategy as the one presented in Vilamala et al. (15), using Deep Convolutional Neural Networks with EEG signals, i.e., multi-channel time series. In comparison to Vilamala et al. (15), we reduce the number of hidden layers from 16 to 4, use a simpler approach for the generation of spectrograms, and do not rely on transfer learning from a network trained on a visual recognition task. Indeed, we believe such a pre-training would initialize the filtering weights to detect object-like features not present in spectrograms. The proposed method outperforms several shallow methods used for comparison as presented in the results section.

Lastly, we employ deep-learning visualization techniques for the interpretation of results. Once a network has been trained, one would like to understand what are the key features it is picking up from the data for classification. We show below how this can be done in the context of EEG spectrogram classification, and how it can be helpful in identifying physiologically meaningful features that would be hard to select by hand. This is also very important for the clinical translation of such techniques, since black-box approaches have been extensively criticized.

## 2. Materials and Methods

### 2.1. Deep Learning in the Spectrogram Representation

Our goal here will be to train a network to classify subjects from the EEG spectrograms recorded at baseline in binary problems, with classification labels such as HC (healthy control), PD (idiopathic RBD who will later convert to PD), etc.

Here we explore first a deep learning approach inspired by recent successes in image classification using deep convolutional neural networks designed to exploit invariances and capture compositional features in the data [see e.g., (9, 11, 12)]. These systems have been largely developed to deal with image data, i.e., 2D arrays, possibly from different channels, or audio data [as in van den Oord et al. (16)], and, more recently, with EEG data as well (15, 17). Thus, inputs to such networks are data cubes (multichannel stacked images). In the same vein, we aimed to work here with the spectrograms of EEG channel data, i.e., 2D time-frequency maps. Such representations represent spectral dynamics as essentially images with the equivalent of image depth provided by multiple available EEG channels (or, e.g., current source density maps or cortically mapped quantities from different spatial locations). Using such representation, we avoid the need to select frequency bands or channels in the process of feature selection. This approach essentially treats EEG channel data as an audio file, and our approach mimics similar uses of deep networks in that domain.

RNNs can also be used to classify images, e.g., using image pixel rows as time series. This is particularly appropriate in the case of the data in this study, given the good performance we obtained using ESNs on temporal spectral data Ruffini et al. (14). We study here also the use of stacked architectures of long-short term memory network (LSTM) or gated-recurrent unit (GRU) cells, which have shown good representational power and can be trained using backpropagation (12, 18, 19).

Our general assumption is that some relevant aspects in EEG data from our datasets are contained in compositional features embedded in the time-frequency representation. This assumption is not unique to our particular classification domain, but should hold of EEG in general. In particular, we expect that deep networks may be able to efficiently learn to identify features in the time-frequency domain associated to bursting events across frequency bands that may help separate classes, as in “bump analysis” (20). Bursting events are hypothesized to be representative of transient synchrony of neural populations, which are known to be affected in neurodegenerative diseases such as Parkinson's or Alzheimer's disease (21).

Finally, we note that in this study we have made no attempt to fully-optimize the network architecture. In particular, no fine-tuning of hyper-parameters has been carried out using a validation set approach, something we leave for future work with larger datasets. Our aim has been to implement a proof of concept of the idea that deep learning approaches can provide value for classification and analysis of time-frequency representations of EEG data—while possibly providing new physiological insights.

### 2.2. Study Subjects

Idiopathic RBD patients (called henceforth RBD for data analysis class labeling) and healthy controls were recruited at the Center for Advanced Research in Sleep Medicine of the Hôpital du Sacrè-Cœur de Montréal as part of another study and kindly provided for this work. The protocol was approved by the Hôpital du Sacré-Cœur de Montréal Ethics Committee, and all participants gave their written informed consent to participate. For more details on the protocol and on the patient population statistics (age and gender distribution, follow up time, etc.), see Rodrigues-Brazéte et al. (7) and Ruffini et al. (8).

**Figure 1:**
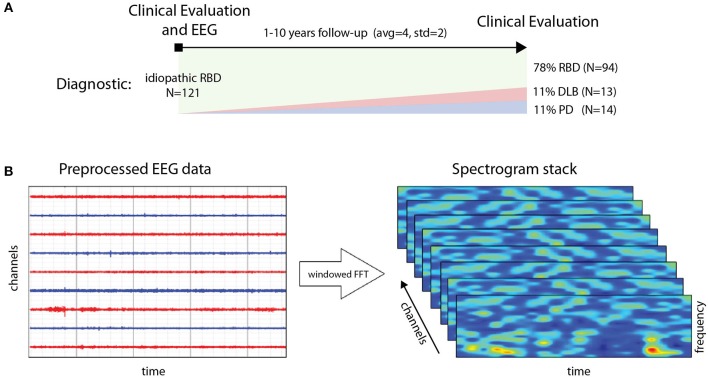
(**A**, top) Generation of spectrogram stack for each data epoch for a subject from preprocessed (artifact rejection, referencing, detrending) EEG data. (**B**, bottom) Timeline and data collection study design: from diagnosis and EEG data collection to follow up with clinical evaluation for conversion to PD and DLB (or remaining idiopathic RBD).

The dataset includes a total of 121 patients diagnosed with idiopathic RBD (of which 118 passed the first quality tests) and 85 healthy controls (of which only 74 provided sufficient quality data) without sleep complaints and in which RBD was excluded. EEG data was collected in every patient at baseline, e.g., when patients were still RBD. After 1–10 years of clinical follow-up 14 RBD patients converted to PD, 13 to DLB, while the rest remained idiopathic RBD (see Figure 1).

In addition to EEG recording at baseline (further described below) participants also underwent a complete neurological examination by a neurologist specialized in movement disorders and a cognitive assessment by a neuropsychologist. The only data used from the follow-up evaluation, which was conducted on average 10 years after baseline, was the updated diagnosis change, if any, from RBD into PD or DLB, or the confirmation of the RBD diagnosis. These data elements have been used here as ground truth in the DCNN training and in the performance evaluation on the test set as set up in the cross validation procedure.

RBD was diagnosed based on AASM Version II (https://aasm.org/aasm-updates-scoring-manual-version-2-2-with-new-option-for-monitoring-respiratory-effort-during-hsat/). This included a history of dream enactment behaviors and a subsequent assessment of overnight polysomnography (PSG) evaluation including video recording and EMG evaluation (22). EEG was acquired at the end of the PSG recording session in awake state.

PD was diagnosed following the Movement Disorder Society Clinical Diagnostic Criteria for Parkinson's disease (PD) (23). In early recordings, the criteria was the standard at that time based on Hughes et al. (24). DLB diagnosis was based on standard procedures described in McKeith et al. (25). Some subjects may have gone through neuroimaging (MRI, as no DAT Scan was available in Canada) for confirmation or differential diagnosis, but not in a systematic way in the overall PD/DLB population.

No healthy controls reported abnormal motor activity during sleep or showed cognitive impairment on neuropsychological testing. Only a subset of healthy controls was followed up. In general, patients were recruited within a year of RBD diagnosis. However, we note as a limitation that the cohort was recruited during a period of 15 years, which may have affected the recruiting conditions.

### 2.3. EEG Dataset

All RBD patients with a full EEG montage for resting-state EEG recording at baseline and with at least one follow-up examination (without EEG) after the baseline visit were included in the study. The first valid EEG for each patient enrolled in the study was considered baseline.

As in related work (7, 8, 14), the raw data in this study consisted of resting-state EEG collected from awake subjects using 14 scalp electrodes. The recording protocol consisted of conditions with periods of with eyes open of variable duration (~2.5 min) followed by periods with eyes closed in which patients were not asked to perform any particular task. EEG signals were digitized with 16-bit resolution at a sampling rate of 256 S/s. The amplification device bandpass filtered the EEG data between 0.3 and 100 Hz with a notch filter at 60 Hz to minimize line power noise. All recordings were referenced to linked ears.

### 2.4. Preprocessing and Generation of Spectrograms

To generate spectrograms (here called frames), EEG data from each channel was processed using Fourier analysis (FFT) after detrending blocks of 1 s with a Hann window (FFT resolution is 2 Hz) (see Figure 1). Twenty second 14 channel artifact-free epochs were collected for each subject, using a sliding window of 1 s. FFT amplitude bins in the band 4–44 Hz were used. The resulting data frames are thus multidimensional arrays of the form [channels (14)] x [FFTbins (21)] x [Epochs (20)]. To avoid biases, the number of frames per subject was fixed as a trade-off between data per subject and number of subjects included, to 148, representing about 2.5 min of data. We selected a minimal suitable number of frames per subject so that each subject provided the same number of frames. For training, datasets were balanced for subjects by random replication of subjects in the class with fewer subjects. For testing, we used a leave-pair-out strategy [LPO, see (26)], with one subject from each class. Thus, both the training and test sets were balanced both in terms of subjects and frames per class. Finally, the data was centered and normalized to unit variance for each frequency and channel.

### 2.5. Network Architectures

**Figure 2:**
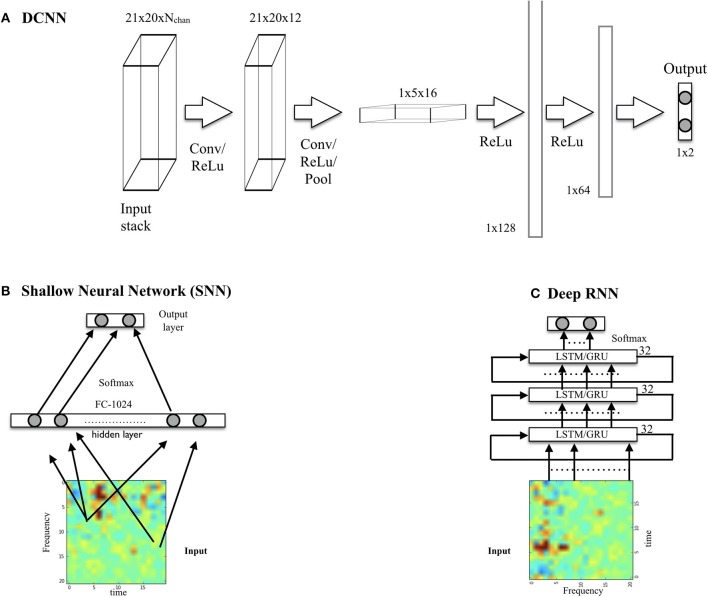
**(A)** DCNN model displaying input, convolution with pooling layers, and hidden-unit layers. The input consists of a spectrogram stack (with a spectrogram per chosen EEG channel). The output, here for the binary classification problem using one-hot encoding, is a two node layer. **(B)** Shallow neural network architecture used for comparison. **(C)** Deep RNN using LSTM or GRU cells.

We have implemented three architectures: DCNN and stacked RNN, as we now describe, plus a shallow architecture for comparison (see Figure 2).

#### 2.5.1. DCNN Architecture

The network (which we call *SpectNet*), implemented in *Tensorflow* (27), is a relatively simple four hidden-layer convolutional net with pooling (see Figure 2). Dropout has been used as the only regularization. All EEG channels may be used in the input cube. The design philosophy has been to enable the network to find local features first and create larger views of data with some temporal (but not frequency) shift invariance via max-pooling.

The network has been trained using a cross-entropy loss function to classify frames (not subjects). It has been evaluated both on frames and, more importantly, on subjects by averaging subject frame scores and choosing the maximal probability class, i.e., using a 50% threshold. For development purposes, we have also tested the performance of this DCNN on a synthetic dataset consisting of Gaussian radial functions randomly placed on the spectrogram time axis but with variable stability in frequency, width and amplitude (i.e, by adding some jitter top these parameters). Frame classification accuracy was high and relatively robust to jitter (~95–100%, depending on parameters), indicating that the network was capable of learning to detect burst-like features with time-translational invariance and frequency specificity.

#### 2.5.2. RNN Architecture

The architectures for the RNNs consisted of stacked LSTM (12, 18) or GRU cells (19). The architecture we describe here consists of three stacked cells, where each cell uses as input the outputs of the previous one. Each cell used 32 hidden units, and dropout was used to regularize it. The performance of LSTM and GRU variants was very similar.

## 3. Results

### 3.1. Classification Performance Assessment

Our goal is to classify subjects (e.g., HC or PD converter labels) rather than frames. The performance of the networks has been evaluated in the balanced dataset using two metrics in a leave-pair out cross-validation framework—where the data from a subject in each class is left out for validation (LPO). First, using the accuracy metric (probability of good a classification), and second, by using the area under the curve (AUC) using the Wilcoxon-Mann-Whitney statistic (26). To map out the classification performance of the DCNN for different parameter sets, we have implemented a set of algorithms based on the *Tensorflow* package (27) as described in the following pseudocode:

 
   REPEAT N times (experiments):
    1- Choose (random, balanced) training and
     test subject sets (leave-pair-out)
    2- Augment smaller set by random
     replication of subjects
    3- Optimize the NN using stochastic
     gradient descent with frames as inputs
    4- Evaluate per-frame performance on
     training and test set
    5- Evaluate per-subject performance
      averaging frame outputs
   END
   Compute mean and standard deviation of
    performances over the N experiments
 

For each frame, the classifier outputs the probability of the frame belonging to each class [using *softmax*, see, e.g., (12)] and, as explained above, after averaging over frames per subject we obtain the probability of the subject belonging to each class. This provides an interesting score in itself. Classification is carried out by choosing the class with maximal probability.

**Figure 3:**
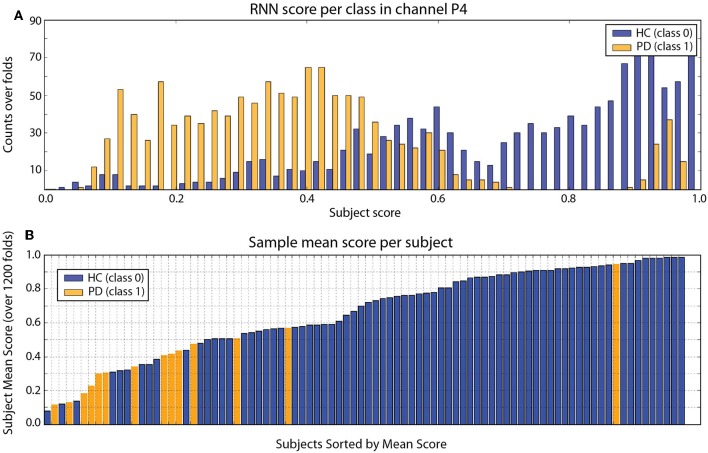
**(A)** RNN Frame score histogram per class (HC in blue, PD in orange) using one channel (P4). **(B)** Subject mean score across folds. In this particular run, with mean ACC = 80%, AUC = 87% (both ±1%). There are clearly some subjects that are not classified correctly (this is consistently with DCNN results). The PD outlier is unusual in terms of other metrics, such as slow2fast ratio (EEG slowing) or LZW complexity (8). Results from the DCNN are very similar.

The results from classification are shown in Table 1 for the HC vs. PD problem and the HC+RBD vs. PD+DLB problem, which includes more data. Sample results for the RNN architecture (which are very similar to DCNN results) are provided in Figure 3. For comparison, using a shallow architecture neural network resulted in about 10% less ACC or AUC (in line with our results using support vector machine (SVM) classifiers (Soria-Frisch et al., in preparation), which required feature selection). On the other hand, in Ruffini et al. (14), a peak accuracy of 85% was reached in the balanced problem of HC vs PD, although this required appropriate feature selection (a selection of channels and bands), and in Ruffini et al. (8) similarly high AUC performance was reached using global (in channel and frequency space) complexity metrics.

**Table 1:** Performance in different problems using a single EEG channel (P4, see Figure 4).

**Problem**	**N train/test**	**Frame train/test ACC**	**Subject test ACC (AUC)**
DCNN: HC vs. PD	2x73 / 2x1	80% / 73%	79% (87%)
RNN: HC vs. PD	2x73 / 2x1	77% / 74%	81% (87%)
DCNN: HC+RBD vs. PD+DLB	2x159 / 2x1	73% / 68%	73% (78%)
RNN: HC+RBD vs. PD+DLB	2x159 / 2x1	76% / 68%	72% (77%)

*From left to right: architecture used and problem addressed (groups); Number of subjects in training and test sets per group (always balanced); train and test average performance on frames; test accuracy and LPO cross-validation area-under-the-curve metric (AUC) (26). Results to ±1%*.

**Figure 4:**
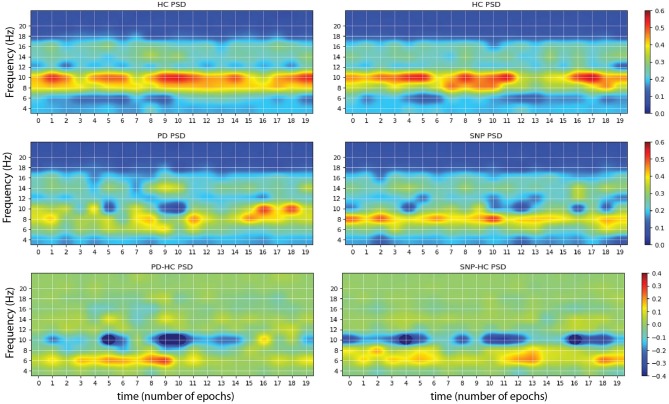
Sample images produced by maximizing network outputs for a given class. **(Left)** From a network was trained using P4 electrode channel data on the problem of HC vs. PD. The main features are the presence of 10 Hz bursts in the image maximizing HC classification **(Top)** compared to more persistent 6 Hz power in the pathological spectrogram **(Middle)**. The difference of the two is displayed at the bottom. **(Right)** Network was trained using P4 electrode data on the problem of HC vs. PD+DLB (i.e., HC vs. RBDs that will develop an α-synucleinopathy or SNP). The main features are the presence of 10 Hz bursts in the HC class maximizing image **(Top)** compared to more persistent 6–8 Hz power bursting in the pathological spectrogram **(Middle)**.

Figure 4 provides the performance in the HC vs. PD problem using different EEG channels (statistics computed using a smaller number of folds).

### 3.2. Interpretation

**Figure 5:**
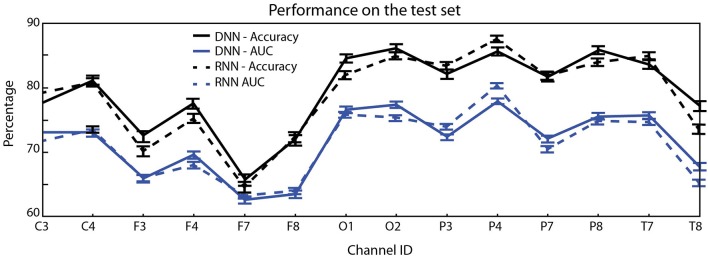
Mean test accuracy (blue) and AUC (black) per EEG channel (averages and standard error of the mean evaluated over 2,000-folds) for the single channel HC vs. PD classification problem. Occipital and parietal electrodes provide better discrimination (top: DCNN architecture, bottom: RNN).

Once a DCNN has been trained, it can be used to explore which inputs optimally excite network nodes, including the output nodes that provide the classification (29). The algorithm for doing the latter consists essentially in maximizing a particular class score using gradient descent, starting from, e.g., a random noise image. An example of the resulting images using the trained DCNN above can be seen in Figure 5, where image corresponds to the input that maximizes each class output, e.g., HC vs. PD. This is a particularly interesting technique in our diagnosis/prognosis problem and provides new insights on the class-specific features in EEG of each class. In the case of a HC vs. PD trained network, we can see alterations in the alpha and theta spectral bands, appearing differentially in the form of bursts in each class. In the difference spectrograms we can observe the disappearance of alpha bursts in exchange with bursting at lower frequencies. This findings are consistent with others relating to alterations and slowing of EEG (6–8, 28, Soria-Frisch et al., in preparation), and in particular of longitudinal alpha frequency and theta frequency band relative power increases in PD with dementia (30). However, they point out in more detail what the network has learned as feature to separate the classes: bursting in the observed bands. This adds a dimension (time) to the usually identified features (power, slowing).

## 4. Discussion

Our results using deep networks are complementary to earlier work using machine learning to analyze this type of data using SVMs and ESNs. However, we deem the use of deep learning methods to be particularly interesting for various reasons. First, they largely mitigate the need for feature selection (in this case the choice of spectral bands and channels). Here we preprocessed the EEG data to obtain spectrograms as a way to simplify the learning task given the limitations in data availability (given enough data, it would seem natural to work with raw or minimally cleaned up multichannel EEG data). Secondly, the employed method represents an improvement over related prior efforts, increasing performance by about 5–10% in AUC (28, Soria-Frisch et al., in preparation).

The obtained results and especially the ones derived from the use of feature visualization are in agreement with the findings of slowing of EEG in PD with respect to HC and RBD patients as observed in previous studies, i.e., power increase in lower frequency bands and decrease in higher ones. More specifically, the shifting of bursting events in the alpha band to lower frequencies is especially interesting and may suggest potential mechanistic explanations regarding the effects of disease on the alpha band underlying circuitry. This underscores the fact that the DCNN can pick up relevant discriminative features without explicitly being tuned to do so, which is not the case for those previous studies with hand-picked features.

The performance of the network was higher with the task of discriminating HC and converters than RBD non-converters and converters, which is expected and probably reflecting different time courses of disease in subjects. This reflects a limitation in our study, namely, that RBD diagnosis and recruitment may have happened along different timepoints for each subject, creating a confound in the analysis.

We note that another limitation in the used dataset is the presence of healthy controls without follow up, which may be a confound for the network—worsening its performance, as some controls may actually be prodromal PD, for example [around 2.2% (31)]. We hope to remedy in the future this by enriching our database with improved diagnosis and follow up methodologies. In addition to dataset quality improvements, future steps include the exploration of this approach with larger datasets as well as a more systematic study of network architecture and regularization schemes. This includes the use of deeper architectures, improved data augmentation methods, alternative data segmentation and normalization schemes. With regard to data preprocessing, we should consider improved spectral estimation using more advanced techniques such as state-space estimation and multitapering—as in Kim et al. (32), and working with cortically or scalp-mapped EEG data prior creation of spectrograms.

Although here, as in Vilamala et al. (15), we worked with time-frequency pre-processed data, the field will undoubtedly steer toward working with raw data in the future when larger datasets become available—as suggested in Schirrmeister et al. (33). Working with time-frequency power representations is definitely a limitation, given current view indicating that neural processing involves both amplitude and phase of signals, e.g., as in communication through coherence or, more generally, oscillation-based communication (34).

In closing, we note that the techniques used in this pilot study can be extended to other EEG related problems, such as brain-computer interfaces, sleep scoring, detection of epileptiform activity or EEG data pre-processing, where the advantages of deep learning approaches may prove useful as well.

## Data Availability

The data analyzed in this study was obtained from Jacques Montplaisir's team at the Center for Advanced Research in Sleep Medicine affiliated to the University of Montréal, Hopital du Sacre-Coeur, Montréal during a previous study. The dataset used for deep learning in this paper (EEG spectrograms) is available upon request to giulio.ruffini@neuroelectrics.com.

## Ethics Statement

Idiopathic RBD patients (called henceforth RBD for data analysis labeling) and healthy controls were recruited at the Center for Advanced Research in Sleep Medicine of the Hôpital du Sacré-Cur de Montréal as part of another study and kindly provided for this work. All patients with a full EEG montage for resting-state EEG recording at baseline and with at least one follow-up examination after the baseline visit were included in the study. The first valid EEG for each patient enrolled in the study was considered baseline. Participants also underwent a complete neurological examination by a neurologist specialized in movement disorders and a cognitive assessment by a neuropsychologist. No controls reported abnormal motor activity during sleep or showed cognitive impairment on neuropsychological testing. The protocol was approved by the hospital's ethics committee, and all participants gave their written informed consent to participate. For more details on the protocol and on the patient population statistics (age and gender 1distribution, follow up time, etc.), see Rodrigues-Brazéte et al. (7) and Ruffini et al. (8).

## Author Contributions

DI and MC preprocessed the EEG data to produce artifact free spectrograms. LD-V contributed to the implementation of the neural networks and revised the manuscript. AS-F contributed to the design of the study, interpretation of the results, and revised the manuscript. J-FG and JM designed the data collection study, collected the EEG data, and revised the manuscript. RP contributed to the study design, data collection, and revision of the manuscript. GR designed the analysis pipeline, implemented the neural networks, analyzed the results, and wrote the manuscript.

### Conflict of Interest Statement

Neuroelectrics and Starlab authors disclose commercial interests in the development of EEG derived biomarkers. GR is a co-founder of Starlab and Neuroelectrics. The remaining authors declare that the research was conducted in the absence of any commercial or financial relationships that could be construed as a potential conflict of interest.
